# Show me your ID: NLR immune receptors with integrated domains in plants

**DOI:** 10.1042/EBC20210084

**Published:** 2022-09-30

**Authors:** Clemence Marchal, Vassiliki A. Michalopoulou, Zhou Zou, Volkan Cevik, Panagiotis F. Sarris

**Affiliations:** 1 The Sainsbury Laboratory, University of East Anglia, Norwich Research Park, NR4 7UH, Norwich, United Kingdom; 2 Institute of Molecular Biology and Biotechnology, Foundation for Research and Technology-Hellas, Heraklion 70013, Crete, Greece; 3 Department of Biology and Biochemistry, The Milner Centre for Evolution, University of Bath, Bath BA2 7AY, United Kingdom; 4 Department of Biology, University of Crete, 714 09 Heraklion, Crete, Greece; 5 Department of Biosciences, College of Life and Environmental Sciences, University of Exeter, Exeter, United Kingdom

**Keywords:** NLR, NLR engineering, NLR-ID, plant immunity

## Abstract

Nucleotide-binding and leucine-rich repeat receptors (NLRs) are intracellular plant immune receptors that recognize pathogen effectors secreted into the plant cell. Canonical NLRs typically contain three conserved domains including a central nucleotide binding (NB-ARC) domain, C-terminal leucine-rich repeats (LRRs) and an N-terminal domain. A subfamily of plant NLRs contain additional noncanonical domain(s) that have potentially evolved from the integration of the effector targets in the canonical NLR structure. These NLRs with extra domains are thus referred to as NLRs with integrated domains (NLR-IDs). Here, we first summarize our current understanding of NLR-ID activation upon effector binding, focusing on the NLR pairs Pik-1/Pik-2, RGA4/RGA5, and RRS1/RPS4. We speculate on their potential oligomerization into resistosomes as it was recently shown for certain canonical plant NLRs. Furthermore, we discuss how our growing understanding of the mode of action of NLR-ID continuously informs engineering approaches to design new resistance specificities in the context of rapidly evolving pathogens.

## Introduction

Plants, unlike mammals, lack an adaptive immune system. Instead, they have evolved two-level innate immunity based on the expression of surface and intracellular receptor proteins [1]. Nucleotide-binding and leucine-rich repeat receptors (NLRs) belong to the second category and recognize secreted pathogens’ molecules known as effectors. This recognition often leads to a programmed cell death in plants known as the Hypersensitive Response (HR) to limit pathogen spread in neighboring cells, tissues, and/or organs [1,2]. NLRs are mostly composed of three conserved domains: a central nucleotide-binding (NB-ARC) domain, C-terminal leucine-rich repeats (LRRs), and an N-terminal domain. Based on the nature of this N-terminal domain, NLRs are broadly categorized into three major subgroups: Toll/interleukin-1 receptor (TIR)-NLRs, Coiled-coil (CC)-NLRs, or Resistance to powdery Mildew 8 (RPW8-like)-NLRs [3]. Plant NLRs recognize pathogen effectors through multiple mode of actions, reviewed in [4] and [5]. A recent evolutionary model [6] proposes that NLRs may have evolved from single units able to both detect and respond to the presence of effectors, referred to as singleton NLRs, to functionally specialized units that either detect (sensor NLRs) or execute the response (helper or executor NLRs) to the presence of the pathogen. Sensor and helper NLRs work in pairs (further described below) or in more complex interconnected networks [7–9].

A subcategory of NLRs carry additional noncanonical domain(s) and can make up to 10–15% of the NLRome of a given plant species [10,11]. Functional studies showed that these extra domains were involved in direct or indirect effector recognition [12–16]. Therefore, it was proposed that NLRs with extra noncanonical domains have evolved from the integration of the effector target in the canonical NLR structure [17]. These NLRs are thus commonly referred to as NLR-IDs (NLR with integrated domain(s)). Kinase, WRKY and zinc-finger BED domains are among the most commonly found IDs [10,11,18,19]. Interestingly, in the case of Pii-2 from rice, the integrated NOI (NO_3_-Induced) domain binds the host protein Exo70-F3, which is a target of the pathogen effector AVR-Pii from *Magnaporthe oryzae* [15]. Pii-2 indirectly recognizes AVR-Pii via Exo70-F3 and it was hypothesized that the original effector target was a NOI-Exo70-F3 complex. Hence, the NOI integration has enabled Pii to monitor *Os*Exo70-F3 and detect AVR-Pii [15]. Several functionally characterized NLR-IDs work in pair with a canonical NLR, where the NLR-ID is the sensor that recognizes the pathogen effector(s) and the canonical NLR is the executor required for the activation of immune response [12–16]. Another characteristic of these NLR pairs is their close proximity in the genome. The pairs regularly appear in a head-to-head orientation, allowing them to share a common promoter for tight control of their expression. However, not all NLR-IDs have a genetically linked canonical NLR in the genome [20–23]. It is unclear whether such NLR-IDs recognize pathogen effector(s) and activate immune response as singletons, or with the help of a genetically unlinked helper. We show a comprehensive list of all cloned and studied NLR-IDs along with their executors, if known, in Table 1.

**Table 1:** A comprehensive list of all cloned and studied NLR-IDs to date, along with their executor NLR and pathogen resistance, where these are known

NLR-ID	Type	ID code	Host species	Integrated domain	Executor NLR	ID code	Resistance	Citation
Adnr1	CNL	TraesCS5A02G344100	*Triticum aestivum* cv. Chinese spring	ANK, WRKY	Adnr1-RGA4 (?)	Traes CS5A02G344000	*Diuraphis noxia*	[83]
*Bn*RPR1	TNL	NA	*Brassica napus*	B3, TFSIIN	*Bn*RPR2	NA	NA	[84]
CHS3	TNL	NP_197291	*Arabidopsis thaliana* ecotype Col-0	LIM, DA1-like domain	CSA1	NP_197290	NA	[85,86]
DAR5	RPW8-like	NP_201464	*A. thaliana* ecotype Col-0	LIM, DA1-like domain	NA	-	NA	[87]
*Os*RPR1	CNL	OsJ_34782	*Oryza sativa Japonica group*	WRKY (x2)	*Os*RPR2	OsJ_34781	NA	[88]
Pi1-5	CNL	AEB00617	*Oryza sativa Indica group* (C101LAC)	AAA	NA	-	*M. oryzae*	[89]
Pi5-3	CNL	Os09g15850	*O. sativa Japonica Group* cv. Nipponbare	Apoptotic protease-activating factors, helical domain	Pi5-1	Os09g15840	NA	[90]
Pi7-J-1	CNL	ASM94220	*O. sativa Indica group* (cv. Jao Hom Nin)	AAA	Pish-J	KY225901.1	*M. oryzae*	[91]
Pia-2 (RGA5)	CNL	AB604627	*O. sativa Japonica Group* cv. Sasanishiki	HMA-like	Pia-1 (RGA4)	AB604622.1	*M. oryzae*	[34]
Pii-2	CNL	QDZ58247	*O. sativa Japonica Group*	NOI	Pii-1	BAN59294	*M. oryzae*	[92]
Pik-1	CNL	ADZ48537	*O. sativa Japonica Group*	AAA, HMA-like (Uniprot)	Pik-2	P0DO07.1	*M. oryzae*	[93]
Pike-1	CNL	NA	*O. sativa Indica group* (C101LAC)	CtNL	Pike-2	NA	*M. oryzae*	[94]
Pik-h1	CNL	AET36549	*O. sativa Japonica Group*	AAA	Pik-h2	AET36550	*M. oryzae*	[95]
Pik-m1	CNL	AB462324	*O. sativa Japonica Group* cv. Tsuyuake	HMA-like (Uniprot)	Pik-m2	D5L9H7	*M. oryzae*	[96]
Pik-p1	CNL	ADV58352	*O. sativa Japonica Group*	AAA	Pik-p2	ADV58351	*M. oryzae*	[24]
Pik-s1	CNL	AET36547	*O. sativa Japonica Group*	HMA-like	Pik-s2	AET36547	*M. oryzae*	[97]
PiPR1	CNL	XM_015780628	*O. sativa Japonica Group* cv. Nipponbare	ZnF_BED	NA	-	*M. oryzae*	[98]
Pi-ta	CNL	ACX94088	*O. sativa Japonica group*	Thioredoxin	NA	-	*M. oryzae*	[99]
RGA2a	CNL	AGQ17376	*Aegilops tauschii*	EXO70	RGA1e	AGQ17384	-	[100]
RGH2	CNL	NA	*Hordeum vulgare* subsp. *vulgare*	EXO70	RGH3	NA	*Blumeria graminis* f. sp. *hordei*	[101]
RLM3_Col	TNL	NM_001341182	*A. thaliana* ecotype Col-0	BRX (3x)	NA	-	*Leptosphaeria maculans, Alternaria brassicicola, Alternaria brassicae, and Botrytis cinerea*	[102]
RPG1	CNL	Q8L3P8	*H. vulgare* subsp. *vulgare*	Pseudokinase domain (pK1), active kinase domain (pK2)	NA	-	*Puccinia graminis* f. sp. *tritici*	[103]
RPG5	CNL	ACH69774	*H. vulgare* subsp. *vulgare*	Serine/Threonine protein kinase	RGA1,RPG4 (Adf2)	ACH69773,ACH69772	*P. graminis f.* sp. *tritici*	[104,105]
Rph15	CNL	KAE8770059	*H. vulgare* subsp. *spontaneum*	ZnF_BED	NA	-	*Puccinia hordei*	[106]
Rpp1-R1	ONL^a^	PRGDB240989 (Glyma.18G280300)	*Glycine max*	ULP1 protease	NA	-	*Phakopsora pachyrhizi*	[107]
Rpp1-R3	ONL^a^	PRGDB236486 (Glyma.18G281500)	*G. max*	ULP1 protease	NA	-	*P. pachyrhizi*	[107]
Rpp1-R4	ONL^a^	PRGDB236494 (Glyma.18G281600)	*G. max*	ULP1 protease	NA	-	*P. pachyrhizi*	[107]
Rpp1-R5	ONL^a^	PRGDB236455 (Glyma.18G281700)	*G. max*	ULP1 protease	NA	-	*P. pachyrhizi*	[107]
RPP2A	TNL	NP_193685	*A. thaliana* ecotype Col-0	AAA (2x)	RPP2B	NP_001328446	*Hyaloperonospora arabidopsidis*	[108]
RRS1B	TNL	NM_180802	*A. thaliana* ecotype Col-0	WRKY	RPS4B	NP_001330960	*Pseudomonas syringae*	[42]
RRS1-R	TNL	HQ170631	*A. thaliana* ecotype Nd-1	WRKY	RPS4	NP_199338	*P. syringae*, *Ralstonia solanacearum*	[109]
RRS1-S	TNL	NM_123894	*A. thaliana* ecotype Col-0	WRKY	RPS4	NP_199338	*P. syringae*	[109]
RRS1-Ws	TNL	AB470471.1	*A. thaliana* ecotype Ws-0	WRKY	RPS4-Ws	AB470473.1	*P. syringae*, *R. solanacearum*, *Colletotrichum higginsianum*	[110]
SLH1	TNL	BAD38678	*A. thaliana* ecotype No-1	WRKY	RPS4	NP_199338	*P. syringae*, *R. solanacearum*	[111]
Ta4ANPR1	ONL^a^	NA	*T. aestivum* cv. Chinese spring	HTH (x2), BTB, ANK (x2), NPR1_like_C	Ta4ANPR1-RGA4	NA	*P. graminis* f. sp. *tritici*	[112]
Ta7ANPR1 (RGA5)	ONL^a^	NA	*T. aestivum* cv. Chinese spring	HTH (x2), BTB, ANK (x2), NPR1_like_C	Ta7ANPR1-RGA4	NA	*P. graminis* f. sp. *tritici*	[112]
Ta7DNPR1	ONL^a^	NA	*T. aestivum* cv. Chinese spring	HTH (x2), BTB, ANK (x2), NPR1_like_C	Ta7DNPR1-RGA4	NA	*P. graminis* f. sp. *tritici*	[112]
TRIDC5AG050380	CNL	TRIDC5AG050380	*Triticum dicoccoides*	ANK, WRKY	NA	-	*Puccinia striiformis* f. sp. *tritici*	[21]
Tsn1	CNL	ADH59425	*Triticum turgidum* subsp. *durum*	Pkinase	NA	-	*Parastagonospora nodorum*, *Pyrenophora triticirepentis*	[113]
WRKY19	TNL	NM_001125496	*A. thaliana* ecotype Col-0	PAH, WRKY (2x), MAPK	DSC1	NP_192938	*Meloidogyne incognita*	[114]
Xa1	CNL	AB002266	*O. sativa Indica group* (strain IR-BB1)	ZnF_BED	NA	-	*Xanthomonas oryzae* pv. *oryzae*	[115]
Xo1	CNL	NA	*Oryza sativa Aromatic Group* cv. Carolina Gold Select	ZnF_BED	NA	-	*X. oryzae pv. oryzae|X. oryzae pv. oryzicola*	[23,116]
Yr5	CNL	QEQ12705	*T. aestivum*	ZnF_BED	NA	-	*P. striiformis* f. sp. *tritici*	[117]
Yr7	CNL	QEQ12704	*T. aestivum*	ZnF_BED	NA	-	*P. striiformis* f. sp. *tritici*	[117]
YrSP	CNL	QEQ12706	*T. aestivum*	ZnF_BED	NA	-	*P. striiformis* f. sp. *tritici*	[117]
YrU1	CNL	QIM55694	*Triticum urartu*	ANK, WRKY	NA	-	*P. striiformis* f. sp. *tritici*	[21]

Most of the NLR-IDs presented in this list have been attached from RefPlantNLR [82].

a Stands for ‘Other’-NL. For NLRs without a CC or TIR domain but a noncanonical domain integrated at their N-terminus (based on RefplantNLR nomenclature [82]).

In this review, we report on the latest advances in the understanding of NLR-ID mode(s) of action and how it continuously informs NLR-ID engineering. We speculate on the activation mechanisms of NLR-IDs in regard to the recently solved structures of plant NLR resistosomes.

## Current understanding of activation mechanisms of NLR-IDs upon effector recognition

Among all characterized NLR-IDs, three NLR pairs have been extensively studied: the rice CNLs conferring resistance against *M. oryzae* RGA5/RGA4 [16] and Pik-1/Pik-2 [24,25] and the RRS1/RPS4 TNLs from *A. thaliana*, conferring resistance against *R. solanacearum*, *P. syringae* pv. *tomato*, and *C. higginsianum* [26–29]. Hence, we focus our report on these three NLR pairs.

### *RGA4*/*RGA5*

RGA4 and RGA5 recognize two sequence-unrelated effectors AVR-Pia and AVR1-CO39 from rice blast pathogen *M. oryzae* [16]. NLR-ID protein RGA5 contains a heavy metal-associated (HMA) domain at the C-terminus after the LRR domain. Interestingly, alternate splicing of RGA5 generates two transcript isoforms that are sequence identical up until the C-terminus where RGA5-A isoform contains the HMA domain, but RGA5-B does not. Only RGA5-A confers resistance to *M. oryzae* isolates expressing AVR-Pia or AVR1-CO39 and interacts with these effectors via its HMA domain [16]. Although AVR-Pia and AVR1-CO39 are sequence-unrelated, they possess highly similar β-sandwich structures that are characteristic of the *M. oryzae* AVRs and ToxB-like (MAX) effector family and bind RGA5^HMA^ at the same interface [30–32]. Furthermore, RGA4 and RGA5 interact through their CC domains and form homo- and hetero-dimers [33]. The NLR protein RGA4 triggers effector independent HR when expressed transiently in both rice protoplasts and in *N. benthamiana*. However, this HR is repressed when RGA4 is co-expressed with RGA5. The present study suggests that upon direct interaction of the effector protein AVR-Pia with the HMA domain in RGA5, repression mediated by RGA5 on RGA4 is relieved and HR occurs [33].

### *Pik-1*/*Pik-2*

The NLR pair Pik-1 and Pik-2 from rice confers resistance to *M. oryzae* following recognition of the effector protein AVR-Pik [25,34,35]. Allelic series were described for both the NLR pair and the effector with Pik alleles showing different recognition specificities to AVR-Pik variants [36–38]. Similar to RGA5, Pik-1 contains an HMA domain but located between CC and NB-ARC domains. Pik-1- and Pik-2-mediated immune activation occurs following direct interaction of AVR-Pik with the HMA domain in Pik-1 [14,38]. AVR-Pik effector proteins share the same MAX fold as AVR-Pia and AVR1-CO39. However, the binding interface of Pikp-1^HMA^/AVR-PikD is different from that of RGA5^HMA^/AVR-Pia or AVR1-CO39 [14,38]. Additionally, a tripartite complex involving Pikp-1, Pikp-2, and AVR-PikD is formed upon effector binding to Pikp-1. This finding suggests a receptors cooperation for the Pikp-1/Pikp-2 pair, rather than a negative regulation as it was reported for the RGA4/RGA5 pair [39]. However, how RGA4 or Pik-2 activates immunity and triggers cell death is only partially understood.

### *RRS1*/*RPS4*

Studies involving *A. thaliana* RRS1/RPS4 immune receptors pair provide further insights into the immune activation by NLR/NLR-ID pairs. RRS1 and RPS4 confer resistance to the bacterial pathogens *P. syringae* and *R. solanacearum* through the recognition of the effectors AvrRps4 and PopP2, respectively [12,13,29,40,41]. In *A. thaliana*, two different RRS1 alleles show different recognition specificities. RRS1-S (in ecotype Col-0) recognizes AvrRps4 but not PopP2, while RRS1-R (in ecotype Ws-2) can recognize both AvrRps4 and PopP2. RPS4 is a canonical TNL type of resistance protein, while RRS1-R is a TNL protein with an integrated WRKY-like domain near its C-terminus. Interestingly, there is a paralogous RPS4B/RRS1B pair in *A. thaliana* (ecotype Ws-2) that also recognizes AvrRps4 but not PopP2. The WRKY domains of RRS1 and RRS1B phylogenetically cluster with different WRKY groups, suggesting an independent integration event [42].

Effector recognition occurs by direct binding of structurally distinct AvrRps4 or PopP2 effectors to the integrated WRKY-like domain [12,13]. The structures of the WRKY-like domain of RRS1 in complex with AvrRps4 or PopP2 indicate that the effectors share a similar binding interface to this ID domain that involves the WRKYGQK DNA-binding motif [43,44]. Additionally, AvrRps4 and *At*WRKY41, a host WRKY transcription factor, share the same binding interface as RRS1^WRKY^/AvrRps4 or PopP2 and effector binding reduces the DNA-binding activity of *At*WRKY41 [43]. This suggests that AvrRps4 promotes virulence via sterically blocking DNA binding of WRKY TFs. Deletion of RRS1-R^WRKY^ triggers a constitutive RPS4-dependent immune activation, suggesting that the RRS1-R^WRKY^ maintains the complex in an inactive state [45]. Intramolecular interactions between the WRKY-like domain and its adjacent domain (named DOM4) are disrupted by AvrRps4 effector binding, which de-represses the complex and leads to an immune response activation [45]. PopP2-mediated de-repression of RRS1-R/RPS4 immune complex is likely different and requires the longer C-terminal extension that is present in RRS1-R but not in RRS1-S [45].

Furthermore, multiple sites within the region harboring C-terminal and the WRKY-like domains of RRS1-R but not RRS1-S are phosphorylated [46]. Phosphorylation occurring at Thr1214 is essential to keep the RRS1-R/RPS4 immune complex at repressed state, while dephosphorylation might release this autoinhibition. Interestingly, the same phosphorylation site is acetylated by PopP2, which might prevent phosphorylation and thus activate the complex [46]. PopP2 but not AvrRps4 responsiveness requires phosphorylation at other sites within the C-terminal region of RRS1-R. De-repression of RRS1-R and RRS1-S by effector binding also triggers proximity between their TIR domains and C termini, releasing RPS4^TIR^ from RRS1^TIR^ inhibition [46]. This could lead to self-association-mediated nicotinamide adenine dinucleotide (NAD+) hydrolase (NADase) activity of the executor RPS4 and activation of downstream immune signalling, as shown for two singleton TNL immune receptors RPP1 and ROQ1 [47,48]. Finally, recent work suggests that there is an extra layer of complexity in the interaction between RRS1-R and RPS4 [49]. The authors showed that RRS1-R enhances the HR mediated by several autoactive RPS4 alleles, but not RRS1-S. Additional biochemical and structural studies will be required to characterize the effect of the RPS4 mutations on its interaction with RRS1 alleles.

## Different sensor/executor interactions for different NLR pairs

**Figure 1:**
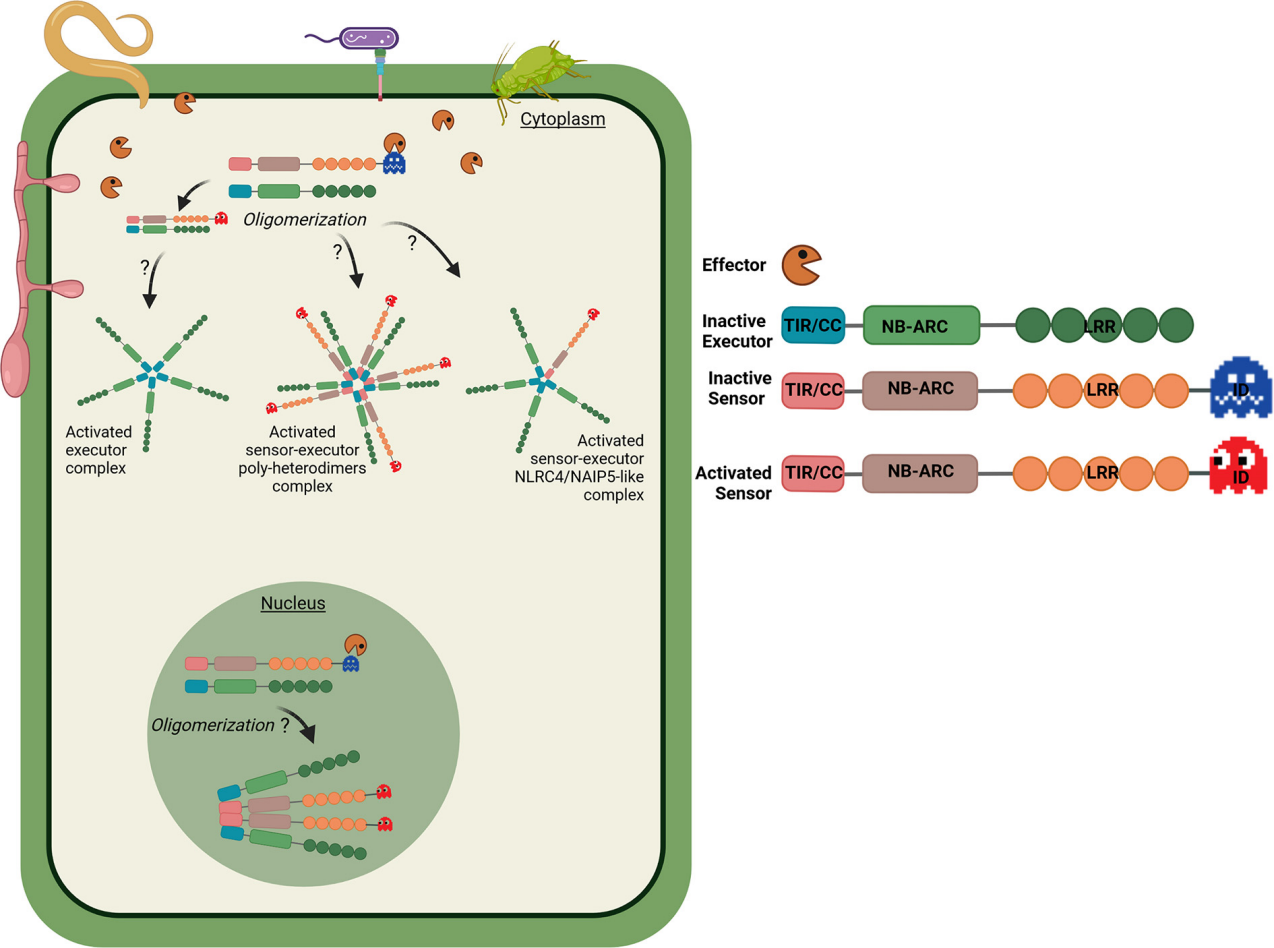
NLR-ID/NLR pair activation model Pathogens such as bacteria, oomycetes, fungi, nematodes, or insects secrete effectors into the plant cytoplasm. The interaction of the effectors with the ID of the sensor NLR, either directly or indirectly, leads to the activation of the sensor and the executor NLRs in the cytoplasm or nucleus. We propose that the activation of the NLR pairs is accompanied with oligomerization of either the executor only, or of the sensor-executor heterodimers or of the sensor-executor in a NLRC4/NAIP5-like complex. The NLR oligomers may vary depending on the nature of the NLR pair. Some NLR pairs may also self-associate in resting stage before their activation by an effector (e.g., Pik-1/Pik-2). Created in BioRender.com.

P-loop (Walker-A) and MHD motifs associated with the NB-ARC domain contribute to NLR activation [50,51]. Although RRS1 and RGA5 both contain a canonical NB-ARC, their p-loop motif is not required for immune response signalling. The same motif, however, is essential for the function of their executors RPS4 and RGA4, respectively [33,45]. This is different from what was observed for Pikp-1 and Pikp-2 receptors, where the p-loop and MHD motifs were required in both sensor and executor for the HR induction in *N. benthamiana* [39]. With the recent demonstration that plant NLRs oligomerize upon activation to form resistosomes [47,48,52,53] that are similar to mammalian inflammasomes [54–57], it would be interesting to see how paired NLRs and NLR-IDs in general fit this model. Are both sensor and executor part of the resistosome? Does the sensor activate/release the executor, which then forms a resistosome? The plant NLR-ID/NLR pairs may function similarly to the mammalian NLRC4/NAIP5 pair that forms a specific inflammasome structure [58,59]. In the plant pairs the sensor NLR-ID interacts with an elicitor (effector), similarly to NAIP5 sensor that interacts with the bacterial flagellin, while the executor NLR is activated (as the NLRC4) and both could potentially form an activated resistosome. Additionally, an activated resistosome containing sensor/executor poly-heterodimers may occur, given that many NLR pairs associate even in the absence of the effector. Alternatively, in the cases where the NLR-ID is not paired, the singleton could be activated by recognizing an elicitor via its ID and form a homo-oligomerized resistosome complex or co-operate with another NLR from a distant genetic locus. A detailed speculation of the formation of such resistosomes in plants is shown in Figure 1. However, these hypotheses remain to be investigated using state of the art structural biology approaches and genetics. Many NLR-IDs have been identified to date (Table 1) and elucidating how effector recognition and activation occur might uncover new mechanisms for this wide NLR subfamily.

## IDs as targets for NLR engineering

**Figure 2:**
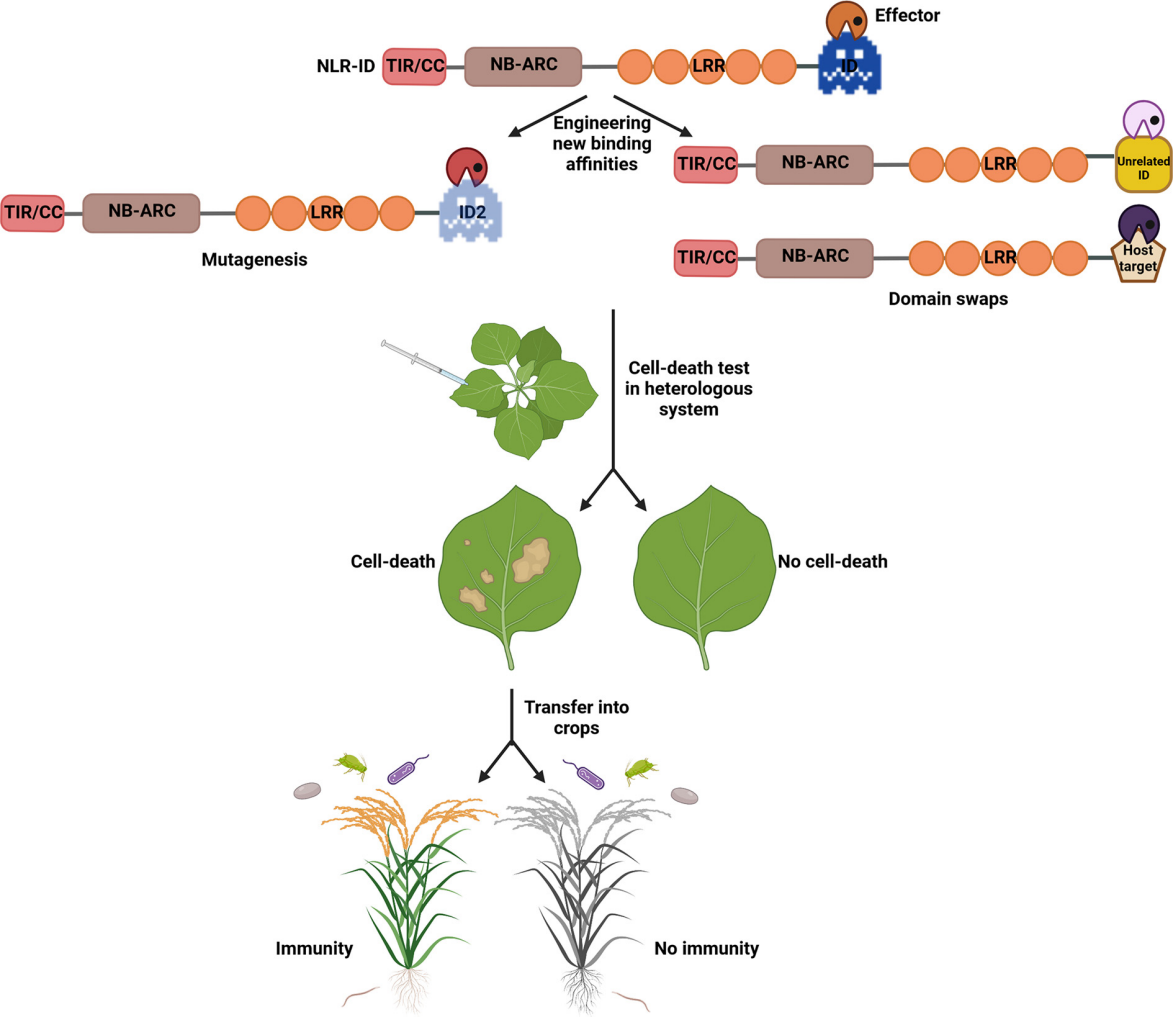
Pipeline to engineer new resistance specificities in NLR-IDs The two main avenues to engineer IDs in NLRs are structure and/or protein–protein interaction-guided mutagenesis or domain swaps. Once new binding affinity is confirmed, the next step is to express engineered sensor, executor, and newly recognized effector in a heterologous system for high-throughput screening. Providing cell death was observed in the presence of the effector, the system can be transferred into crops to test for resistance against pathogen(s), expressing the newly recognized effector. Created in BioRender.com.

Plant pathogens rapidly evolve virulent races that can nullify new resistance specificities shortly after being deployed in the field. To address this, we need to continuously identify new resistance genes and alleles targeting the pathogen genotypes associated with plant disease outbreaks and transfer these into commercial elite cultivars in a timely fashion. With our growing understanding of NLR mode of action, fine-tuning resistance mediated by characterized NLRs and adapting it to current epidemics might become possible. Recent work showed that engineering new resistance specificities in NLR-IDs is achievable, although it involves considerable prior knowledge of the system. The two main avenues for engineering IDs in NLR-IDs are mutagenesis or domain shuffling (Table 2 and Figure 2).

**Table 2:** Examples of NLR-ID engineering with corresponding targets and outcomes

System	Cognate effector(s)	New target(s)	Methods	Gain of binding	HR in *N. benthamiana*	Resistance in stable lines
Pikp-1/Pikp-2	AVR-PikD	AVR-PikD,E,A,C	Structure-guided mutagenesis	Y	Y (not for AVR-PikC)	Not tested
RGA5/RGA4	AVR1-CO39, AVR-Pia	AVR-PikD	Structure-guided mutagenesis	Y	Y	N
RGA5/RGA4	AVR1-CO39, AVR-Pia	AVR-Pib	Structure-guided mutagenesis	Y	Y	Y
RRS1-R/RPS4	PopP2, AvrRps4	SAP05	Domain shuffling	Y	Y	N
Pikm-1/Pikm-2	AVR-PikD,E,A	GFP, mCherry	Domain shuffling	Not tested	Y	Not tested

## Structure-guided engineering of the integrated HMA domain in Pik-1 and RGA5

The integrated HMA domains of rice NLRs Pik-1 and RGA5 were engineered using structure-guided mutagenesis to expand their recognition spectrum or switch effector specificity. De la Concepcion et al. [60] combined favorable binding interfaces from Pikp-1^HMA^/AVR-PikD [14] and Pikm-1^HMA^/AVR-PikD, E, A [38] complexes to design a Pikp-1^HMA^ variant able to bind AVR-PikD, E, A, C and trigger HR in the presence of AVR-PikD, E, A in *N. benthamiana* when co-expressed with its executor Pikp-2. Cesari et al. [61] engineered RGA5^HMA^ (aka Pia-2 ^HMA^), recognizing AVR-Pia and AVR1-CO39, to bind AVR-PikD. The authors combined the binding interfaces of RGA5^HMA^/AVR1-CO39 and Pikp-1^HMA^/AVR-PikD into RGA5^HMA^ to design a RGA5^HMA^ variant able to bind AVR-PikD and retain binding to AVR1-CO39 and AVR-Pia. Co-expressing this RGA5 variant with its executor RGA4 in the presence of Avr-PikD, Avr-Pia or AVR1-CO39 triggers HR in *N. benthamiana*. However, the engineered Avr-PikD recognition did not translate into resistance in rice transgenics infected with *M. oryzae* strains expressing this effector. Finally, Liu et al. [62] modified the HMA domain of RGA5 to gain binding to AVR-Pib by comparing the structure of the RGA5^HMA^/AVR1-CO39 complex to the modelled structure of RGA5^HMA^/Avr-Pib [63]. The engineered RGA5^HMA^ lost AVR-Pia binding and recognition and triggers HR in responses to AVR-Pib in the presence of RGA4 in *N. benthamiana*. Additionally, RGA5^HMA^ variant/RGA4 conferred resistance against *M. oryzae* expressing AVR-Pib in rice transgenics.

## Integrated domain swap in RRS1R and Pik-1

Effector/host target interactions could provide valuable information on how to engineer new resistance specificities to bait effectors [43,64]. Maidment et al. [64] and Oikawa et al. [65] showed that AVR-Pik variants bind rice proteins containing a HMA domain that is phylogenetically related to the HMA domains integrated in Pik-1 alleles and RGA5. All known AVR-Pik variants bind *Os*HIPP19, including the two variants AVR-PikC and F that are not recognized by any known Pik alleles. The structural information of the *Os*HIPP19/AVR-Pik complexes resolved in the present study could thus inform on HMA domain engineering to expand Pik alleles spectrum of recognition to these AVR-Pik variants. Recently, Wang et al. [66] developed a tripartite system to engineer phytoplasma effector SAP05 recognition by the RRS1/RPS4 system. SAP05 mediates the degradation of its targets SPL and GATA transcription factors by hijacking the 26S plant ubiquitin receptor RPN10 [67]. The authors used an autoactive version of RRS1-R, RRS1-R*^slh1^*, RPS4, and RRS1-R fused with the GATA domain that is recognized by SAP05 in this system. In the absence of SAP05, RRS1-R*^slh1^* autoactivity is repressed by RRS1-R-GATA. In the presence of SAP05, RRS1-R-GATA is degraded, re-enabling RRS1-R*^slh1^* autoactivity that is dependent on RPS4. The tripartite system was able to induce HR in the transient system of *N. benthamiana* in the presence of the SAP05 effector. Transgenic Arabidopsis lines expressing RRS1-R-GATA, RRS1-R*^slh1^*, and RPS4 showed delayed symptoms in the presence of the pathogens but were not fully resistant, however.

More recently, Kourelis et al. [68] swapped the HMA domain of Pikm-1 for single variable domain heavy chain (VHH) antibodies (or nanobodies) targeting GFP or mCherry. The Pikm-1-Nanobody fusions (Pikobodies) trigger HR in *N. benthamiana* in the presence of Pikm-2 and the corresponding fluorescent protein. Furthermore, Pikobodies conferred resistance against a Potato Virus X variant expressing free GFP or free mCherry. It remains to be shown whether Pikobody-mediated resistance is functional in stable transgenics, however. Given that nanobodies can be raised against virtually any molecule, Pikobodies have the potential to generate resistance against all major plant pathogens and pests secreting effectors in the plant cell.

## Effector binding does not always translate into immunity

Challenges can arise at each step of the NLR editing process (Figure 2). For example, mutations and/or domain swapping can trigger an autoimmune response when transiently expressed in heterologous systems [37,66,69]. Further fine-tuning of the NLR scaffold is often necessary to prevent this. Additionally, even though new binding affinity can be engineered, binding alone does not automatically translate into an immune response (Table 2 and Figure 2). This is especially true when information on the isolated ID in complex with the effector is used to guide engineering, as it is impossible to predict the effect of the mutation(s) in the context of the full-length receptor. Finally, even if there is an immune response in heterologous systems and transient assays, synthetic NLRs might still not be functional in other systems under stable expression (Table 2 and Figure 2). These hurdles are not impossible to overcome, however. The examples of NLR-ID engineering discussed above illustrate that NLRs can tolerate such edits and even function with non-plant domains. Certain NLR scaffolds may be more tolerant than others to domain swapping and/or targeted edits. For example, orthologues across cereals of RGA5 carry different integrated domains at their C-terminus [18]. Could this mean that RGA5 orthologues might accept domain swaps more easily than other NLR scaffolds? This remains to be investigated.

An additional perspective connected to NLR-IDs is the identification of essential host’s components that are targeted by pathogens and pests to promote susceptibility. All the identified and studied IDs potentially reflect the original targets of the pathogens at the subcellular level so far. This indicates that these integrated domains can be used as a toolbox toward the identification of the original effector targets in the host cells as well as novel host susceptibility components that could be utilized further [70,71].

## Outlook

Combined with the increasing quality of genome assemblies and annotations available for a wide range of plant species and varieties, NLR discovery pipelines [72–74], improved protocols for crop transformation [75,76], and shorter generation times [77,78] and synthetic NLRs will be a valuable addition to our toolkit to design future crops. While developing new resistance specificities might be facilitated in the future, synthetic NLRs will face the same issue as any dominant resistant genes deployed as a single unit. Stewardship plans will thus need to be in place to deploy synthetic NLRs in combination and/or rotation with other resistance genes to prevent rapid resistance breakdown. Additionally, diagnostic methods such as field pathogenomics [79,80] or Marple [81] can identify pathogen isolates causing outbreaks and thus inform targeted engineering of new resistances.

## Summary

- NLR-IDs are present across the plant kingdom.
- Functional studies showed that these extra domains are involved in direct or indirect effector recognition.
- Most functionally characterized NLR-IDs work in pairs.
- It is still unclear how NLR-IDs are activated upon effector recognition.
- Integrated domains in NLRs can be engineered to expand or modify their spectrum of effector recognition.

## Abbreviations

CC: Coiled-Coil
AVR: AVirulent Factor
HMA: Heavy Metal-Associated
HR: Hypersensitive Response
IDs: Integrated Domains
LRRs: Leucine-Rich Repeats
MAX: *M. oryzae* AVRs and ToxB-like
NAD+: Nicotinamide Adenine Dinucleotide
NADase: Nicotinamide Adenine Dinucleotide hydrolase
NB-ARC: Nucleotide Binding domain present in **A**PAF-1 (apoptotic protease-activating factor-1), **R** proteins and **C**ED-4 (Caenorhabditis elegans death-4 protein)
NLRs: Nucleotide-binding and Leucine-rich Repeat receptors
NOI: NO3-Induced
RPW8: Resistance to Powdery Mildew 8
TIR: Toll/Interleukin-1 Receptor
TNL: TIR-containing NLR
VHH: single variable domain heavy chain
WRKY TFs: WRKY Transcription Factors
GFP: Green Fluorescent Protein
MHD: Methionine (M)-Histidine (H)-Aspartate (D) motif
